# The juggling paradigm: a novel social neuroscience approach to identify neuropsychophysiological markers of team mental models

**DOI:** 10.3389/fpsyg.2015.00799

**Published:** 2015-06-11

**Authors:** Edson Filho, Maurizio Bertollo, Claudio Robazza, Silvia Comani

**Affiliations:** ^1^School of Psychology, University of Central Lancashire, Preston, UK; ^2^Behavioral Imaging and Neural Dynamics Center, University “G. d’Annunzio” of Chieti-Pescara, Chieti, Italy; ^3^Department of Medicine and Aging Sciences, University “G. d’Annunzio” of Chieti-Pescara, Chieti, Italy; ^4^Department of Neuroscience, Imaging and Clinical Sciences, University “G. d’Annunzio” of Chieti-Pescara, Chieti, Italy

**Keywords:** team mental models, shared mental models, complementary mental models, juggling, neuropsychophysiology, hyperbrains, social neuroscience

## Abstract

Since the discovery of the mirror neuron system in the 1980s, little, if any, research has been devoted to the study of interactive motor tasks (Goldman, 2012). Scientists interested in the neuropsychophysiological markers of joint motor action have relied on observation paradigms and passive tasks rather than dynamic paradigms and interactive tasks (Konvalinka and Roepstorff, 2012). Within this research scenario, we introduce a novel research paradigm that uses cooperative juggling as a platform to capture peripheral (e.g., skin conductance, breathing and heart rates, electromyographic signals) and central neuropsychophysiological (e.g., functional connectivity within and between brains) markers underlying the notion of team mental models (TMM). We discuss the epistemological and theoretical grounds of a cooperative juggling paradigm, and propose testable hypotheses on neuropsychophysiological markers underlying TMM. Furthermore, we present key methodological concerns that may influence peripheral responses as well as single and hyperbrain network configurations during joint motor action. Preliminary findings of the paradigm are highlighted. We conclude by delineating avenues for future research.

## Introduction

Across domains of human interest, science has always evolved through research paradigms (Kuhn, 1962). In sport science and performance psychology, neuropsychophysiological research has been primarily shaped by the *expert-novice paradigm* (Eklund and Tenenbaum, 2014). Scholars have aimed to identify the neuropsychophysiological markers (i.e., neural and physiological markers associated with psychological constructs) that distinguish high-performing individuals (“experts”) from their low-performing counterparts (“novices”), and optimal (e.g., “flow-feeling”) from poor (e.g., “choke”) performance states (Yarrow et al., 2009; Bertollo et al., 2013). Although various technological methodologies (e.g., fMRI, PET, NIRS, TMS) have been used to study motor tasks, most of what is known about the neuropsychophysiological markers of skilled performance is derived from electroencephalography (EEG) studies in precision sports, such as archery and pistol shooting (Hatfield and Kerick, 2007).

While the study of self-paced sports using EEG has evolved our knowledge of the neuropsychophysiological markers and networks of individuals’ skilled motor performance (Nakata et al., 2010), little is known about the neuropsychophysiological networks involved in successful interactive team actions (Reed et al., 2006; Tognoli, 2008). Since the discovery of the mirror neuron system in the early 1980s, little, if any, research has focused on interactive motor tasks (Goldman, 2012; Schilbach et al., 2013). Furthermore, social biology and social neuroscience researchers have primarily relied on passive (i.e., information flows unidirectionally from an active to a disengaged subject, such as avatars) rather than interactive paradigms (information flows multi-directionally between two or more active individuals; see Di Paolo and De Jaegher, 2012; Konvalinka and Roepstorff, 2012). Our perspective herein is to propose a novel paradigm, using cooperative juggling as a platform, to identify peripheral and central neuropsychophysiological mechanisms underlying the conceptual notion of team mental models (TMM).

We start by providing support for a juggling paradigm. The theoretical roots of TMM, illustrating how a juggling paradigm can advance research on TMM, are then presented. Next, we elaborate on a series of methodological considerations needed to advance our understanding of the neuropsychophysiological markers of TMM. We conclude by presenting preliminary findings and commenting on avenues of future research.

## The Case for a Juggling Paradigm

There is cross-disciplinary evidence that juggling offers a robust platform to advance knowledge in a variety of research domains, including motor behavior, brain sciences, and mathematics (Draganski et al., 2004; Dessing et al., 2012). Our proposal is aimed at identifying the peripheral and central physiological markers of team actions in general, and joint motor actions in particular. We propose that a juggling paradigm can greatly advance knowledge of “multi-brain” interactions during joint motor actions, akin to how research on self-paced sports was used to advance our knowledge of “single brains.” Cooperative juggling presents epistemological and methodological advantages that might help in the identification of neuropsychophysiological markers underlying TMM.

From an epistemological standpoint, cooperative juggling establishes that the locus of interest is on a given team, as two or more jugglers share the goal of “keeping the balls in the air.” To become a team a group of individuals should share a common goal (Carron et al., 2007). Without a shared goal, an assembly of individuals is a “group” rather than a “team.” Moreover, *social loafing* (i.e., a decrease in personal effort when individuals work in groups) is unlikely to occur in cooperative juggling given that individual mistakes and lack of effort are visible to the self and others. With a shared goal and clear performance expectations, the search for the neuropsychophysiological markers underlying TMM and other team-level phenomena (e.g., cohesion, collective-efficacy) is epistemologically valid within a juggling paradigm.

From a methodological standpoint, cooperative juggling represents an interactive task, in the sense that information flows multi-directionally between two or more jugglers. Most existing studies have been based on passive paradigms and cognitive task-analysis (e.g., card playing; music), thus limiting scholars’ ability to ask and respond to questions on joint motor interactions (Schilbach et al., 2013). For instance, in card playing one must play first so that another player has the opportunity to respond. Studies in music have considered musicians playing their own instrument rather than interacting through shared instruments (Lindenberger et al., 2009; Müller et al., 2013). Recent technological advancements, particularly mobile EEG synchronized with kinematic recording devices, allow for the reliable and multimodal monitoring of complex motor actions, including joint actions in cooperative juggling (Reis et al., 2014; Schack et al., 2014). Altogether, we posit that cooperative juggling offers an ideal epistemological and methodological platform to advance the theory of TMM.

## Theoretical Considerations

**FIGURE 1:**
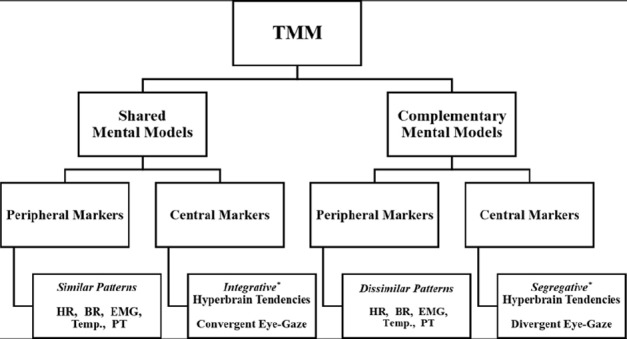
**Proposed hypothetical neuropsychophysiological markers of TMM.** HR, heart rate; BR, breathing rate; EMG, electromyography; Temp., temperature; PT, posture. *Prevalent Topological Configuration between two or more brains.

Team expertise has been associated with the development of team-level cognitive schemas or TMM (Mathieu et al., 2000; Mohammed et al., 2010). To date, however, there is no reliable neuropsychophysiological evidence that team-level cognition exists. In this section, we provide an overview of the concept of TMM, while advancing several testable hypotheses to assess peripheral and central neuropsychophysiological markers of TMM (Figure 1).

### Team Mental Models

The term TMM has been defined as “task and team relevant knowledge that team members bring to a situation” (Cooke et al., 2000, p. 153). While a unified theory of TMM is not available to date (Filho et al., 2015), scholars concur that TMM represent two main forms of “task and team relevant knowledge,” namely *shared* and *complementary* mental models (Cannon-Bowers and Salas, 2001; Mohammed et al., 2010). Shared mental models refer to *communal schemas* about team tasks, strategies, and teammates’ characteristics. Complementary mental models pertain to *idiosyncratic schemas* held by teammates about team tasks, strategies, and teammates’ characteristics (Xinwen et al., 2006). To be successful, a team needs both shared and complementary mental models (Cannon-Bowers and Salas, 2001; Mohammed et al., 2010). Without shared knowledge, teammates cannot develop heuristic routes to facilitate team coordination and optimize decision-making under high-pressure situations (Bearman et al., 2010; Filho and Tenenbaum, 2012). Without complementary knowledge, teammates are unable to compensate for coordination breakdowns or generate creative solutions (Mohammed et al., 2010; Filho and Tenenbaum, 2012).

The existence of shared and complementary mental models has been established through the observation of coordination mechanisms (Cannon-Bowers et al., 1993; Mohammed et al., 2010). From a socio-cognitive standpoint, coordination refers to spatio-temporal synchronized action and effort among teammates and includes (a) explicit coordination, manifested through spoken verbal communication; and (b) implicit coordination, exhibited through non-verbal behavior (Filho and Tenenbaum, 2012). An abundance of research on explicit coordination exists (Mohammed et al., 2010). However, the neuropsychophysiological markers of implicit coordination remain understudied, especially in real-time interactive tasks (Reed et al., 2006; Schilbach et al., 2013). Accordingly, we focus on how the theoretical notions of shared and complementary mental models can be related to peripheral and central neuropsychophysiological variables.

### Peripheral Neuropsychophysiological Markers of TMM

**FIGURE 2:**
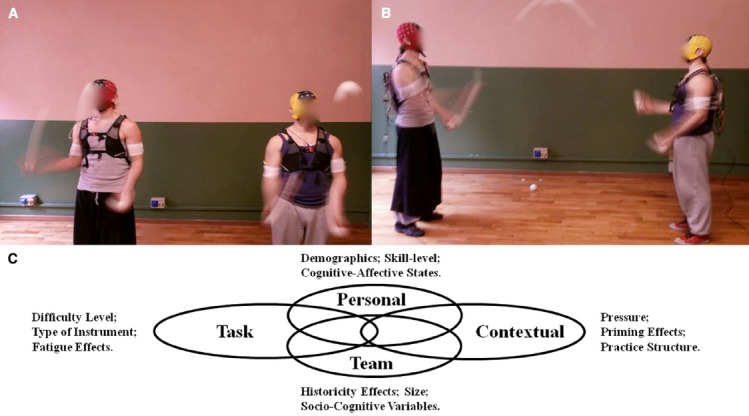
**Illustration of EEG acquisitions in the individual condition of solo juggling (A) and using a cooperative juggling paradigm (B).** Venn Diagram illustrating personal, contextual, task, and team-level factors to be experimentally manipulated and accounted for **(C)**.

To reliably identify neuropsychophysiological markers of TMM, a “control condition” must be defined. In juggling, a control condition would consist of a “solo juggling” (i.e., individual) task to be contrasted with an “interactive” condition (i.e., two or more jugglers established a cooperative interaction by juggling balls with each other) as illustrated in Figures 2A,B, respectively. The absence of a control condition prevents the researcher from being able to identify differences between individual and coupled peripheral and central neuropsychophysiological responses. This rationale is equivalent to current praxis in social neuroscience, where non-clinical individuals are used as controls in studies about social brain disorders (Harris, 2003). The solo and interactive conditions must be similar in terms of *difficulty level*, as established by the same number of degrees of freedom (i.e., the number of balls juggled minus the number of hands). Based on this rationale, if the interactive team-level task is defined as “dyadic juggling with five balls in the cascade style,” then the control condition would consist of “solo juggling with three balls in the cascade style.” Variations in the number of balls can occur as long as the solo and interactive conditions remain comparable. The *difficulty level* can also be established by quantitative (e.g., regression models), survey (e.g., Rates of Perceived Effort), and qualitative (e.g., interview with performers) methods.

A reliable control condition allows for testing of hypotheses. The first hypothesis is that teammates’ neuropsychophysiological responses (Figure 1) should differ in interactive team-level tasks in comparison to individually performed tasks. This difference is due to the coordination effort needed for cooperative work in team settings (Eccles and Tenenbaum, 2004). Although conceptually appealing, this hypothesis has yet to be examined from a neuropsychophysiological standpoint. The second hypothesis is that similar patterns among peripheral neuropsychophysiological responses of teammates performing an interactive motor task might be indicative of shared mental models. This would be in line with the “mimicry coordination mechanism,” which is at the core of Theory of Mind and has greatly influenced research on team processes (Goldman, 2012). Conversely, dissimilar patterns of peripheral neuropsychophysiological responses might be indicative of compensatory activations aimed at reaching team coordination, similar to the notion of complementary mental models.

Conclusions about the relationship of shared and complementary inter-individual neuropsychophysiological patterns should be drawn with care, as these patterns might also be due to task characteristics or the reaching of a physiological plateau. Noteworthy, non-linear functions may also signal TMM, as teammates’ compensatory behaviors and bio-psycho-social states are not necessarily linear processes (Carron et al., 2007). For instance, teammates’ idiosyncratic rhythms, as indicated by the lack of linear or non-linear relationship among paired neuropsychophysiological responses, may signal either complementary mental models (e.g., teammates’ responses are related to task) or the absence of TMM (e.g., teammates’ responses are unrelated to task).

The interpretation of teammates’ coupled neuropsychophysiological patterns should be made in light of previous research on the mind-body connection. For instance, heart rate patterns have been linked to cognitive load and attentional control (Veltman and Gaillard, 1998; Bertollo et al., 2013), whereas breathing pattern is considered an indicator of motor coordination for skills of differing complexity levels (Martin-Harris, 2006; Seifert et al., 2010). Moreover, electromyography (EMG) and posture data can be used to inform research on TMM. Grounded on the notion of mirror neurons, jugglers exhibiting markedly similar EMG waves (forms, intensity, and frequency) in a given muscle group, while leaning toward the same location, may be relying on shared mental models. Conversely, jugglers displaying different EMG activations and distinct yet action-related compensatory posture may be relying on complementary mental models. Measures of hormones in blood plasma, temperature and skin conductance may also help to establish whether teammates share a similar emotional state. For instance, cortisol levels, low temperature on body extremities, and reduced skin conductance have been associated with stress responses (Eklund and Tenenbaum, 2014).

### Central Neuropsychophysiological Markers of TMM

Electroencephalography is commonly considered the most reliable method for studying interactive brains during motor tasks (Konvalinka and Roepstorff, 2012). In this context, functional connectivity and efficiency measures are particularly suitable for the study of joint actions (Sänger et al., 2013). Functional connectivity maps can quantify the functional interdependencies related to shared or complementary mental models. Efficiency measures, such as those provided in Graph Theory, may help to reveal, through a hyperbrain approach, between-brains functional network topologies related to shared or complementary mental models.

The first hypothesis on central markers of TMM would test the notion that each individual possesses idiosyncratic neural functional patterns (complementary mental models) related to the interactive motor task. For instance, a highly skilled juggler may exhibit higher neural efficiency than a less skilled juggler. To this extent, eye-tracking technology could add information on the behavioral basis for the skill level of each juggler. Indeed, fixation and duration of eye-gaze have been linked to central mnemonic adaptations and associated with skill level (Eklund and Tenenbaum, 2014), with experts exhibiting context control (e.g., gaze at a central location) and novices showing target control (e.g., following “targets”) strategies.

The second hypothesis is that two brains engaged in a joint action should show unique systemic and communication characteristics, in comparison to the individual control condition. We would expect that *integrative hyperbrain patterns* (i.e., shared activity among brain cortices) reflect shared mental models, whereas *segregative brain tendencies* (i.e., low hyperbrain functional connectivity) indicate complementary mental models. Altogether, hyperbrain analysis performed through Graph Theory allows for the identification of functional flexibility or *meta-stability* (both integrative and segregative tendencies) of multi-brain networks (Tognoli, 2008), which in turn can serve as a neural index of individual preferences and team expertise. It has long been noted in Gestalt Psychology that a team is “greater than the sum of its parts” (TMM > Σ individuals’ mental models), or “only as strong as its weakest link” (TMM ≤ Σ individuals’ mental models). A hyperbrain approach applied to cooperative juggling can ultimately advance our knowledge of team expertise in interactive tasks by providing evidence of possible dynamic links between two interactive brains. Further manipulating the personal, task, contextual, and team-level factors may help to identify the ensemble of neuropsychophysiological markers of team expertise.

## Methodological Considerations

A methodological cornerstone pertains to the synchronization of two or more acquisition systems used to record neuropsychophysiological signals of joint motor action. Without precisely synchronized systems, it is impossible to reliably identify peripheral and central markers of joint motor action. As opposed to large-scale nomothetic studies, an idiographic approach through a series of well-controlled case studies might be the most appropriate design given that each cooperative team may have a unique “modus operandi.” Furthermore, case studies and small-n studies are appropriate when data acquisition is complex, costly and time intensive, and when potential participants are rare (Editorial Nature Neuroscience, 2004).

Other methodological aspects pertain to *personal*, *task*, *contextual*, and *team-level* factors (Figure 2C). The *person-task-context* notion has been the basis of studies in human action, as per the well-established Action Theory (Schack and Hackfort, 2007). Over the past 30 years, scholars have manipulated personal, task, and contextual variables in the search for answers about skilled movement action (Schmidt and Lee, 2011). Additionally, it is important to account for variance on team-level factors when conducting socio-cognitive research (Feltz et al., 2008). Therefore, we expand on the *personal*, *task*, *contextual*, and *team-level* factors that can be manipulated to advance knowledge of the neuropsychophysiological markers of TMM within a juggling paradigm.

### Personal Factors

*Demographic variables* (e.g., age, gender, hand dominance) should be accounted for as such variables influence performance in individual and team-level actions (Carron et al., 2007). *Skill level* is also likely to influence joint motor action (Eccles and Tenenbaum, 2004). For instance, during cooperative juggling, an expert may have to compensate for mistakes from a novice juggler. Furthermore, *cognitive* (e.g., self-efficacy; associative-dissociative focus) and *affective states* (e.g., arousal and pleasantness) have been associated with performance in motor tasks (Hanin, 2007). Single-item measures, the most ecologically valid approach for collecting data during motor task, can be used to assess the aforementioned factors (Kamata et al., 2002).

### Task Factors

Task *difficulty* can be manipulated by increasing the number of elements to be juggled (easy, moderate, hard levels). Furthermore, changing the juggling instrument *type* (e.g., balls, clubs, diablo) should activate different neuropsychophysiological mechanisms (Beek and Lewbel, 1995). A juggling dyad can be proficient juggling with balls and only mediocre juggling with clubs. Finally, it is important to control for *fatigue effects* that can influence performance and the reliability of the assessment of neuropsychophysiological markers (Marcora and Staiano, 2010).

### Contextual Factors

Manipulating *pressure* through different means (e.g., audience effects; panel of judges) can be used to explore neuropsychophysiological changes (Schilbach et al., 2013). One could explore whether hyperbrain networks change under pressure in comparison to a non-pressure condition. Further, *priming effects* influence a range of social actions (Kuzyakov et al., 2000). Priming positive and negative emotions about a context or unknown juggling partner may induce neuropsychophysiological changes that can affect the interactive motor action. Additionally, manipulating *practice structure* (e.g., blocked or random practice; see Schmidt and Lee, 2011) can advance knowledge on “team learning.” The assumption is that the quality and quantity of TMM can be influenced by practice structure.

### Team-Level Factors

Controlling for *historicity effects* is essential in social interaction studies. The existence, nature and extent of previous interactions influence team processes (Di Paolo and De Jaegher, 2012). Furthermore, the *size of the team* influences team dynamics and performance (Carron et al., 2007). Dyadic teams represent realistic target samples, with larger teams adding complexity to data collection and analysis. *Socio-cognitive variables*, such as cohesion and collective-efficacy, can be used as triangulation sources to help interpret neuropsychophysiological markers of TMM (Filho et al., 2015).

## Preliminary Findings and Avenues for Future Research

The juggling paradigm proposed herein has been implemented in two case studies using two different cooperative juggling dyads and two different experimental conditions: “individual” and “interactive” tasks. In study-1 we targeted peripheral markers (i.e., breathing and heart rate). In study-2 we targeted central markers of TMM by using two synchronized EEG systems. Results from study-1 revealed a strong correlation between the jugglers’ heart rate (*r* = 0.87, *p* < 0.01) and breathing rate (*r* = 0.77, *p* < 0.01) in the interactive condition. Results from study-2, based on a graph theoretical approach, suggested that the juggling dyad presented a hyperbrain pattern that varied with task difficulty, wherein higher values of “small-world-ness” were observed for an easier task in both theta (0.84) and alpha bands (0.82), as compared to a harder task (theta = 0.56; alpha = 0.52). That is, easier tasks fostered more integrative hyperbrain tendencies (shared models), whereas harder tasks elicited more segregative (complementary models) hyperbrain tendencies.

Future research should aim to answer three main questions. First, what are the neuropsychophysiological markers of TMM? Second, how do potential neuropsychophysiological markers of TMM vary in respect to personal, task, contextual, and team-level factors? Third, are changes (i.e., learning) in shared and complementary mental models observable through neuropsychophysiological longitudinal monitoring of cooperative dyads practicing together? The influence of co-regulation training on both functional (integrative and segregative brain tendencies) and structural (neuroplasticity; see Draganski et al., 2004) neurological adaptations should also be advanced. Beyond cooperative tasks, hyperbrain research in competitive tasks may advance knowledge on broader meta-cognitive concepts, including anticipation skills in sports, “strategic mindreading” (Game Theory), and “collective-consciousness.”

## Author Contributions

All authors have contributed with their specific expertise to designing the juggling paradigm presented herein.

### Conflict of Interest Statement

The authors declare that the research was conducted in the absence of any commercial or financial relationships that could be construed as a potential conflict of interest.
